# Development and Pilot Test of a Novel Digital Social Support Intervention for Reducing Hazardous Alcohol Use

**DOI:** 10.1111/acer.70301

**Published:** 2026-04-23

**Authors:** Li Yan McCurdy, Tammy J. Chung, Adam M. Stryjewski, Tara L. Spitzen, Charla Nich, Suchitra Krishnan‐Sarin, Grace Kong, Brian D. Kiluk, Marc N. Potenza

**Affiliations:** ^1^ Department of Psychiatry Yale School of Medicine New Haven Connecticut USA; ^2^ Child Study Center Yale School of Medicine New Haven Connecticut USA; ^3^ Department of Neuroscience Yale School of Medicine New Haven Connecticut USA; ^4^ Wu Tsai Institute Yale University New Haven Connecticut USA; ^5^ Connecticut Mental Health Center New Haven Connecticut USA; ^6^ Connecticut Council on Problem Gambling Wethersfield Connecticut USA

**Keywords:** addiction recovery, digital intervention, hazardous alcohol use, peer support, social support

## Abstract

**Background:**

Community‐based recovery organizations (e.g., mutual‐help groups) can be a potent source of social support that facilitates addiction recovery. To increase awareness of and engagement with these organizations, we developed a digital intervention called *Let's Do Addiction Recovery Together!* (LDART) for individuals who want to reduce their hazardous alcohol use. LDART encourages daily goal‐setting, provides motivational video messages from peers, and provides information on recovery organizations.

**Methods:**

We conducted a single‐arm study to evaluate feasibility, acceptability, and preliminary efficacy of LDART. Thirty adults (mean age 44.5 years old, 53.8% women) who wanted to reduce their hazardous alcohol use were invited to use LDART for 28 nights. Participants completed an acceptability survey post‐intervention, and a subset were interviewed to provide additional feedback. Measures of recovery organization engagement and alcohol use were collected pre‐intervention, post‐intervention, and at 1‐month post‐intervention and summarized using descriptive statistics.

**Results:**

The recruitment rate was 10 participants per month, and 76.7% of enrolled participants completed the post‐treatment assessment. On average, participants accessed LDART on 22.9 out of 28 nights (81.8%). Most participants (81.0%) reported that they would recommend LDART to someone trying to cut down or quit drinking. Participants self‐reported more recovery organization engagement and less alcohol consumption during the month they used LDART and the month after completing LDART, relative to the month prior to using LDART.

**Conclusions:**

LDART was well‐received by adults wanting to reduce drinking, as determined by engagement rates with LDART and acceptability ratings. Preliminary efficacy results warrant further testing of this intervention as a way to increase engagement with recovery organizations and decrease hazardous alcohol use.

## Graphical abstract

A digital intervention called *Let's Do Addiction Recovery Together!* (LDART) was developed to encourage goal‐setting, provide motivational messages from peers, and provide information on recovery groups to individuals in early recovery. LDART was well‐received, and preliminary results suggest that LDART may increase engagement with recovery groups and decrease alcohol use.

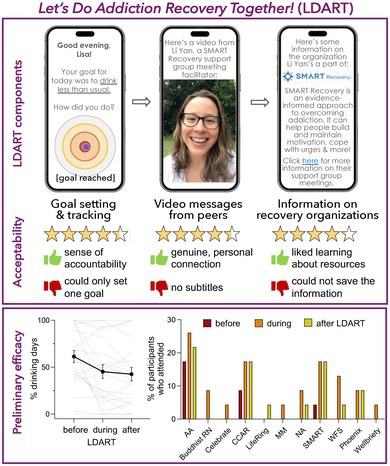

## 1 Introduction

Problematic alcohol use is associated with multiple physical and mental health concerns (Griswold et al. 2018), yet recovery is possible (Kelly et al. 2017). The National Institute on Alcohol Abuse and Alcoholism recognizes that recovery is often associated with improvements in well‐being, such as enhancements in social support, and that “improvement in these domains may, in turn, promote sustained recovery” (Hagman et al. 2022). Indeed, social support is associated with better recovery outcomes such as lower relapse likelihood (Maisto et al. 2024; Sliedrecht et al. 2019), particularly when support is provided by recovery‐related social networks (Andreas et al. 2010; Beattie 2001; Beattie and Longabaugh 1999; Brooks et al. 2017; Groh et al. 2007; Liu et al. 2017; Longabaugh et al. 2010; Stevens et al. 2015; Stout et al. 2012; Westwell et al. 2024). Such support can be provided by community‐based addiction recovery organizations (CROs) such as the 12‐step group Alcoholics Anonymous. We use the term CROs here to refer to non‐clinical organizations that provide freely and publicly available recovery‐related services, often via peers with lived experience of addiction and recovery. CROs provide invaluable support for people in recovery and may facilitate recovery through multiple mechanisms such as improving recovery capital (i.e., internal and external resources needed to overcome addiction), increasing self‐efficacy, and increasing recovery‐related social networks (Kaskutas et al. 2002; Kelly 2017; Kelly, Stout, et al. 2011; Kelly, Urbanoski, et al. 2011; Morgenstern et al. 1997).

Although 12‐step programs are arguably the most well‐known CROs, there are several other CROs that exist in person and/or online. These include other mutual‐help groups (e.g., SMART Recovery, Moderation Management, Women for Sobriety), recovery community centers (e.g., Connecticut Community for Addiction Recovery, or CCAR) and sober active communities (e.g., The Phoenix), whose evidence base for sustaining recovery continues to grow (Beck et al. 2017, 2025; Brooks et al. 2017; Heinrich et al. 2025; Islam et al. 2023; Kelly et al. 2017; Liu et al. 2020; Moos and Timko 2008; Patterson et al. 2025; White 2009; Zemore et al. 2017, 2018). These CROs cater to a variety of demographic, cultural, and religious affiliations, and may be a better “fit” than 12‐step programs for some people (Kelly et al. 2024, 2023; Kelly and White 2012), but may be less well‐known and thus underutilized (Beck et al. 2025; Kelly et al. 2017).

Efforts have been made to increase engagement with CROs. Referral to community resources is often done via passive methods such as providing pamphlets or handouts about CROs, which has limited effectiveness (Hogue et al. 2024). Another set of interventions involves trained personnel (e.g., clinicians, peers in recovery) providing “assertive linkages” or “warm handoffs” between individuals in recovery and CROs by providing information about CROs, arranging for individuals to meet people from the CRO, and following up with individuals to determine whether they engaged with the CRO (Best et al. 2017; Manning et al. 2012; Timko et al. 2011). While these interventions are often more engaging and effective (Richter et al. 2016), the reliance on trained personnel may limit scalability. Additionally, most existing interventions are designed to link to one specific CRO (e.g., Alcoholics Anonymous).

To fill a gap in the addiction intervention space, we sought to develop an engaging and scalable digital intervention that increases awareness of and engagement with multiple CROs. Our intervention, called *Let's Do Addiction Recovery Together!* (LDART), It is a web‐based program that provides daily motivational messages and information on a variety of CROs. To increase the relatability and approachability of the CROs, information on CROs is provided alongside pre‐recorded motivational video messages from people associated with the CROs. The video messages are presented in a goal‐setting context, where participants set and report on daily recovery goals (e.g., to drink less than usual, to engage with a CRO). Overall, LDART is a social support intervention designed to reduce hazardous alcohol use by increasing one's recovery‐specific social support network and one's sense of perceived social support (Hogan et al. 2002).

LDART is grounded in social cognitive theory (SCT), which emphasizes the importance of beliefs about the self, human agency, and social influences on behavioral change (Bandura 1991). Indeed, Bandura himself posited that problematic substance use is “a social problem, not just a personal problem” (Bandura 1999). SCT has four constructs associated with behavior change: *goal setting* (e.g., setting specific and achievable objectives for reducing alcohol use), *self‐efficacy* (e.g., the belief in one's ability to reduce one's alcohol use), *outcome expectations* (e.g., the anticipated positive consequences of reducing one's alcohol use), and *socio‐cultural factors* (i.e., environmental influences that may facilitate or hinder one's efforts to reduce alcohol use) (Bandura 2004). LDART's goal‐setting component aligns directly with the SCT construct of *goals* as a direct mechanism of behavior change, and we propose that goal‐setting, specifically reaching the goals that one sets for themselves, also increases *self‐efficacy* via *personal mastery* (Bandura 2001, 2004). The messages from peers are proposed to increase *self‐efficacy* via *vicarious experience* and *verbal persuasion*, as well as to create positive *outcome expectancies* (in the form of positive, supportive, and encouraging messages) associated with making efforts to reach recovery goals (Bandura 2004). Finally, information on CROs is proposed to improve *socio‐structural factors* (Bandura 2004) by increasing awareness of and engagement with sources of social support in one's community.

In this manuscript, we describe the development of the therapeutic components of LDART and its theoretical basis in SCT. We also present results from a single‐arm pilot study evaluating its feasibility, acceptability, and preliminary efficacy among 30 adults who wanted to reduce their heavy drinking. Our primary outcomes were related to study feasibility in terms of recruitment and retention rate, and intervention acceptability assessed using quantitative and qualitative approaches. Since this was a pilot study, no feasibility or acceptability benchmarks were set a priori. We hypothesized that participants would use LDART with or without compensation for using it and that most participants would recommend LDART to someone who wanted to reduce their heavy drinking. We also explored preliminary efficacy and hypothesized that decreases in alcohol use and improvements in SCT‐based mechanisms of behavior change, such as self‐efficacy, and socio‐structural factors, such as engagement with CROs, would be observed.

## 2 Materials and Methods

### 2.1 Study Design

This study was approved by Yale University's Institutional Review Board (HIC#2000035434) and was registered on ClinicalTrials.gov (NCT06022107). This was a single‐arm pilot study using a longitudinal within‐subjects design. Participants completed baseline assessments prior to using the intervention for 28 days. They then completed follow‐up assessments at the end of the 28‐day period (i.e., post‐intervention) and again at 1‐month post‐intervention. A subset of participants completed a qualitative interview with the study team within a month of completing the final assessment.

### 2.2 Participants

Participants were recruited from January to April 2024. Participants were recruited using flyers posted in common areas at outpatient addiction treatment facilities and recovery community centers and electronic flyers emailed by the Yale Center for Clinical Investigation to people who expressed interest in participating in research. To be eligible for study participation, individuals had to: (1) be at least 18 years of age, (2) reside in Connecticut, USA, at the time of study participation, (3) be able to read and understand English at a sixth‐grade reading level or higher, (4) have a past‐year Alcohol Use Disorders Identification Test (AUDIT) score of 8 or more, indicating hazardous or more severe alcohol use (Saunders et al. 1993), (5) report at least one heavy drinking day in the past month, defined as consuming more than three or four drinks for women and men, respectively, in one sitting, and (6) self‐report a desire to cut down or quit using alcohol. Exclusion criteria included having a vulnerable population status (e.g., pregnant people and prisoners) or receiving inpatient psychiatric treatment at the time of study participation.

### 2.3 Procedures

Procedural details can be found in the study protocol manuscript previously published (McCurdy et al. 2023). Upon establishing eligibility through a Qualtrics screen, each participant was scheduled for a 30‐min study visit with a member of the study team, conducted remotely (i.e., over the videoconferencing platform, Zoom). During this visit, participants received a brief description of the study, a walk‐through demonstration of how to use LDART, and completed informed consent with an electronic signature. After the visit, participants completed the baseline assessment, described in Section 2.5.

After completing the assessment, participants received a unique link for accessing LDART that contained a brief video tutorial and were instructed to begin using LDART within a week. Participants received compensation for use of LDART on the first and third weeks of the intervention ($2 for each night that they used LDART or $15 if they used LDART on all seven nights in the week); no compensation was provided for using LDART during the second or fourth week. Monetary incentives were used in this study to increase engagement with the intervention, and they were alternated across weeks to determine the effect of monetary compensation on intervention engagement, as has been done in other studies (Griffith Fillipo et al. 2022).

Participants were asked to complete follow‐up assessments via a Qualtrics link at the end of the 28‐day period (i.e., post‐intervention) and again at 1‐month post‐intervention (described in Section 2.5.2.2). Participants received a $30 gift card for each completed survey. Ten participants were randomly selected for a 30‐min qualitative interview on Zoom to gain additional insight into acceptability and were compensated with a $20 gift card.

### 2.4 Intervention

**FIGURE 1:**
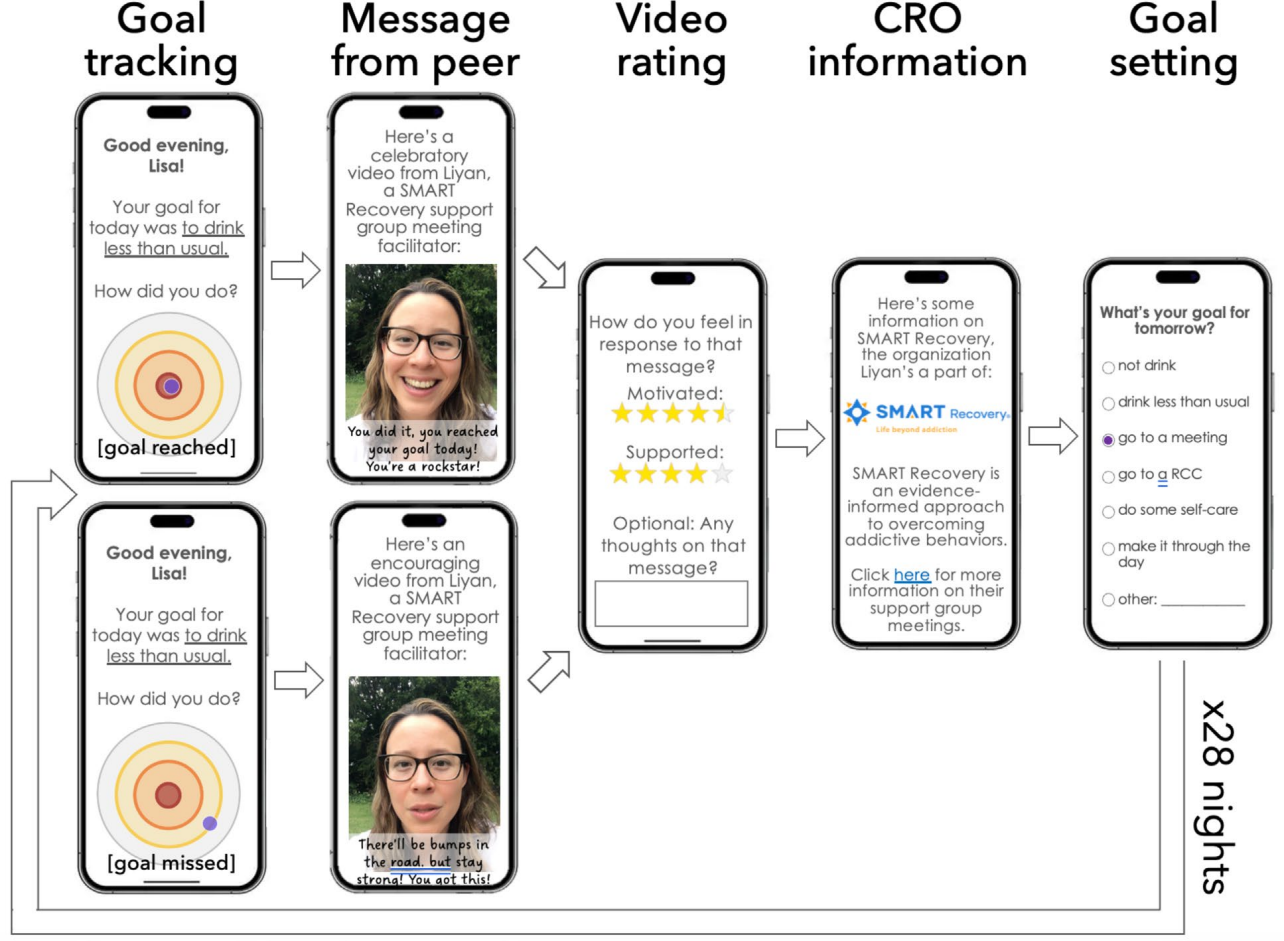
LDART user flow diagram. Representative examples of screenshots of what participants would view each night, depending on whether they reached their daily recovery goal. Images of the first author are used in place of individuals associated with CROs to maintain their confidentiality. Example transcripts are overlayed on the videos in this figure to provide examples of what is said in each video type. Note that participants could select from up to 10 goals, but only 7 are depicted here for ease of display.

LDART is a web‐based intervention which can be accessed via any device that has internet access. The user flow diagram depicting examples of the screens displayed to participants is provided in Figure 1. Each night for 4 weeks, approximately an hour before their typical bedtime, each participant received a text and/or email notification to access LDART. On the LDART website, participants were reminded of the recovery goal they had set for themselves the night before (e.g., drink less than usual, engage with a CRO). They indicated whether they had reached their goal by clicking on a dartboard: within or outside of the bullseye if they had reached or missed their goal, respectively. They then received a message from a peer associated with a CRO, which correspondingly either congratulated them for having reached their goal or encouraged them to keep trying if they had missed. On most nights, this message was in the form of a pre‐recorded video; due to the limited number of videos, participants received written messages (which were composites of the video transcripts) on the remaining nights. (More details are available in Supporting Inforamtion). Participants then indicated how motivated and supported they felt in response to the message, on a scale of 0–5 stars (where 0 and 5 were the lowest and highest scores, respectively). They then received information about the CRO with whom the person in the video was associated; a list of CROs featured in LDART is provided in Table S1. Participants were then prompted to set a goal for the following day. The entire process of using LDART took a few minutes each night. Details about each component of LDART and its theoretical justification are included in Supporting Inforamtion, and an overview of the theoretical framework linking specific LDART components with SCT constructs is provided in Figure S1.

### 2.5 Measures

#### 2.5.1 Baseline Measures

Participants completed a questionnaire assessing demographic characteristics and access to electronic devices. Severity of alcohol use was assessed using the AUDIT (Saunders et al. 1993). Additionally, participants were asked to respond to the following questions: “At what age did you first start drinking regularly?” and “How many years do you think you have had problems with drinking?” Participants were also given a list of CROs and asked to indicate which CROs they had heard of and with which they had engaged (e.g., attended a meeting) at least once in their lifetime.

#### 2.5.2 Primary Outcomes

##### 2.5.2.1 Feasibility

Study feasibility was assessed in terms of recruitment and retention. Recruitment feasibility was measured as the number of adults who consented to study participation divided by the number of months of trial recruitment (Pfledderer et al. 2024); this is a measure of recruitment efficiency that is sometimes referred to as “accrual rate” (Michaeli et al. 2024) or “recruitment rate” (Jacques et al. 2022). Retention feasibility was calculated as the number of participants who completed the study (i.e., completed the 1‐month post‐intervention assessment) divided by the number of participants who enrolled in the study (Pfledderer et al. 2024).

##### 2.5.2.2 Acceptability

Acceptability was assessed using three metrics. First, engagement with the intervention, defined as the number of nights (out of 28) participants had used LDART, was used as a measure of acceptability (Pfledderer et al. 2024). Since compensation for using LDART was provided in weeks 1 and 3 but not in weeks 2 and 4, additional analyses were conducted to compare engagement during weeks that were compensated versus not compensated.

Second, acceptability was measured with a 9‐item self‐report acceptability assessment questionnaire that was completed within a week of completing LDART, modeled after previous questionnaires by the study team (Kong et al. 2017). Participants responded to a series of statements regarding the helpfulness of each LDART component and perceptions on the frequency of engaging with the intervention. The list of statements can be found in Table 2. Each statement was rated on a 5‐point Likert scale, where 1 indicated “strongly disagree,” and 5 indicated “strongly agree.” There were also two open‐ended questions for participants to describe what they liked about the intervention and make suggestions for improvement.

Third, to contextualize the quantitative acceptability results, 10 participants were randomly selected for a follow‐up 30‐min qualitative interview to identify which components of LDART they did and did not like. Interviews were conducted after participants completed the final follow‐up assessment. These selected participants did not differ from the larger baseline sample for most demographic characteristics (Table S2), except that the former group had a higher proportion of men and began regular alcohol use at a younger age than the latter group. There were no differences in engagement with LDART or acceptability ratings between the two groups (Table S3).

#### 2.5.3 Secondary Outcomes

All secondary outcomes were collected at three time points: up to a week prior to beginning using LDART, within a week post‐intervention, and approximately 1‐month post‐intervention.

##### 2.5.3.1 Alcohol Consumption

Alcohol consumption was quantified using Timeline Follow‐back (Sobell and Sobell 1992), a validated, self‐reported approach that provides specific dates for alcohol use in the past 28 days and the number of standard drinks consumed on each drinking day. *Percent Drinking Days* was calculated as the percentage of days in the 28‐day window in which one or more standard drinks were consumed. *Percent Heavy Drinking Days* was calculated as the percentage of days in the 28‐day window in which four (if female) / five (if male) or more standard drinks were consumed (National Institute on Alcohol Abuse and Alcoholism 2004).

##### 2.5.3.2 CRO Engagement

*CRO engagement* refers to the self‐reported number of hours spent engaging with CROs (i.e., attending support group meetings, engaging in recovery community center activities, and participating in sober active community activities) in the past 28‐day time window. Participants first indicated with which CROs they had engaged in the past 28 days and then were prompted to estimate how many hours they had spent with each organization in the past 28 days. A binary version of this variable was created to reflect whether each participant had engaged with any CROs in the past 28 days. *Number of CROs engaged with* refers to the number of different types of CROs participants utilized in the past 28‐day time window: for example, a participant who attended a SMART Recovery support group meeting, a cross‐fit activity organized by the Phoenix, and a crafts session at CCAR would have engaged with three CROs.

##### 2.5.3.3 Self‐Efficacy

*Self‐efficacy* was measured using the General Self‐Efficacy Scale (Schwarzer et al. 1995). This is a 10‐item questionnaire comprising statements such as, “I can remain calm when facing difficulties because I can rely on my coping abilities.” Each question is rated on a four‐point scale, where the total score ranges between 10 and 40, with higher scores reflecting greater self‐efficacy. The General Self‐Efficacy Scale was selected over domain‐specific measures of self‐efficacy (DiClemente et al. 1994) because we posited that the goal‐setting component of LDART would lead to increases in self‐efficacy that are specific to the goals that participants set for themselves. For example, participants who repeatedly set and achieve a custom goal such as “go exercise today” might experience increases in exercise self‐efficacy. As such, a general measure was selected to capture potential increases in self‐efficacy across multiple domains.

##### 2.5.3.4 Recovery Capital

*Recovery capital* was assessed using the Brief Assessment of Recovery Capital (BARC; Vilsaint et al. 2017), a 10‐item self‐report measure with 10 domains (substance use and sobriety, global psychological health, global physical health, citizenship and community involvement, social support, meaningful activities, housing and safety, risk‐taking, coping and life functioning, and recovery experience). Each item is rated on a scale of 1–6 (1: strongly disagree, 6: strongly agree), with a range of 10–60, where higher values reflect more recovery capital.

##### 2.5.3.5 Quality of Life

The World Health Organization Quality of Life—Brief Version (WHOQOL‐BREF; World Health 2004) is a 26‐item questionnaire with four domains (score range in parentheses): physical health (7–35), psychological health (6–30), social relationships (3–15), and environment (8–40). Each question is rated on a five‐point Likert scale, where higher values reflect a better quality of life. Each domain score was transformed based on the lowest possible score to convert each domain score into a range from 0 to 100 (Vahedi 2010).

### 2.6 Analytic Strategy

Statistical analyses were conducted in Prism 9 (GraphPad Software, MA, USA). Where relevant, data were tested for normality using the Shapiro–Wilk test. Measures of feasibility and acceptability were quantified using descriptive statistics. The number of days logged onto LDART each week (i.e., engagement) was quantified using a general linear model, and Dunnett's post hoc test was used to determine significant differences in subsequent weeks relative to week 1. To determine whether the number of days that participants accessed LDART each week was significantly greater than zero, the Wilcoxon signed rank test was used, as the data were not normally distributed. To determine the effect of compensation on engagement with LDART, a paired *t*‐test was used to compare total days logged on during weeks 1 and 3 (when compensation was provided) versus weeks 2 and 4 (when compensation was not provided). To compare demographic and acceptability data between participants who completed the qualitative interview and participants who did not, unpaired *t*‐tests or Mann–Whitney tests were conducted with data that were normally and non‐normally distributed, respectively. Chi‐squared (χ^2^) tests were performed to determine differences in proportions.

To analyze the qualitative data, audio recordings of the qualitative interviews were automatically transcribed verbatim using Zoom and verified manually against the audio recordings by a member of the study team (LYM). The interview guide and coding of themes were focused on identifying components of LDART that participants did and did not like. Two coders (TJC & AMS) coded all respondents' answers based on each LDART component. This generated four codes: “goal‐setting,” “messages,” “CRO information,” and “other thoughts.” They first coded answers from two of the same participants to verify coding fidelity, then coded the remaining four transcripts each. After merging their files to create a single document with responses to each of the four codes from all 10 transcripts, a third coder (LYM) summarized these responses, where similar responses were placed in the same category. Filler words, false starts, and repetitions were removed from quotations featured in this manuscript for ease of reading.

For the preliminary efficacy data, participants who did not have data at all three time points were excluded from analyses. As this was a pilot study that was insufficiently powered to determine effect sizes, no statistical analyses were conducted to infer the significance of changes in outcome variables over time. The means and standard deviations of the outcome variables at the three time points were reported and should be interpreted as exploratory. Graphs were plotted in Prism 9 (GraphPad Software, MA, USA). All error bars indicate the standard error of the mean.

## 3 Results

### 3.1 Study Feasibility

**FIGURE 2:**
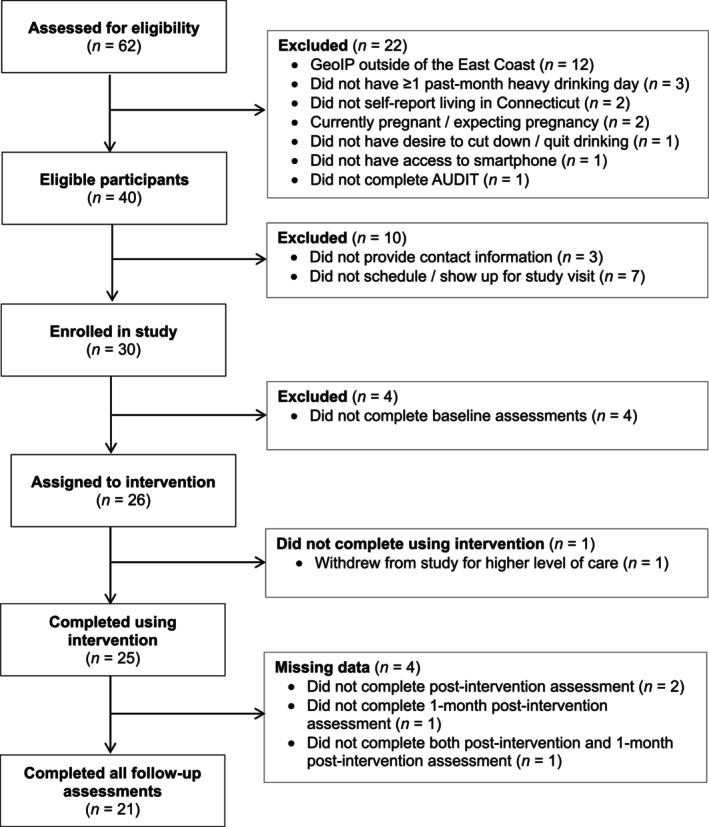
Modified Consolidated Standards of Reporting Trials (CONSORT) diagram.

Participant flow is depicted in Figure 2. Thirty participants were enrolled over 3 months at an approximate recruitment rate of 10 participants per month. All eligible participants who met with the researcher for the study visit enrolled in the study. Of the 26 participants who completed baseline assessments and began using LDART, one participant withdrew from the study after 1 week of using LDART due to the need for a higher level of care. All 25 remaining participants used LDART at least once per week for 4 weeks. The post‐intervention questionnaire and 1‐month post‐intervention questionnaire were completed by 22 and 23 participants, respectively. Thus, the retention rate was 23/30 participants (76.7%).

Even though all 26 participants who completed baseline assessments reported having at least one past‐month heavy drinking day at the time of screening, six participants no longer had at least one past‐month heavy drinking day at the start of using LDART. Analyses excluding these six participants are included in Supporting Inforamtion.

### 3.2 Participant Characteristics

Demographic information of the 26 participants who completed the baseline assessments revealed that participants were mostly non‐Hispanic White adults in their 40s, with a nearly equal number of women and men (Table 1). Most (21/26, 80.7%) participants had a past‐year AUDIT score of 15 or more, indicating a likelihood of moderate‐to‐severe alcohol use disorder. Participants had 17.5 (SD = 8.9) drinking days and 8.6 (SD = 8.9) heavy drinking days on average in the past month. At baseline, participants were familiar with 2 CROs on average, with most participants (16/25, 64.0%) having only heard of 12‐step programs (i.e., Alcoholics Anonymous and/or Narcotics Anonymous). More than half of the participants (15/25, 60.0%) had never attended a CRO meeting/event in their lifetime.

**TABLE 1:** Participant characteristics.

Demographic measures	
Age in years, mean (SD)	44.5 (10.6)
Gender, *n* (%)	
Female	14 (53.8)
Male	12 (46.2)
Race, *n* (%)	
American Indian/Alaskan Native	1 (3.8)
Black/African‐American	6 (23.1)
White/European‐American	18 (69.2)
Prefer not to answer	1 (3.8)
Ethnicity, *n* (%)	
Hispanic/Latinx	2 (7.7)
Not Hispanic/Latinx	24 (92.3)
Education, *n* (%)	
High school or less	2 (7.7)
Some college	10 (38.5)
Associate's, Bachelor's or above	14 (53.8)
Income, *n* (%)	
Less than $50,000	12 (46.2)
$50,000–$99,999	8 (30.8)
More than $100,000	6 (23.1)
Employment, *n* (%)	
Working full‐time	12 (46.2)
Working part‐time	4 (15.4)
Stay‐at‐home parent	1 (3.8)
Retired/Unemployed	6 (23.1)
Other/Prefer not to answer	3 (11.5)
Access to devices, *n* (%)	
Android phone	12 (46.2)
iPhone	15 (57.7)
iPad	5 (19.2)
Laptop/Computer	17 (65.4)
Alcohol measures–mean (SD)	
Years of problematic alcohol use	12.8 (11.4)
Age began regular alcohol use	23.0 (11.5)
Past‐year AUDIT score	22.7 (8.7)
Past‐month drinking days	17.5 (8.9)
Past‐month heavy drinking days	8.6 (8.9)

*Note:* *n* = 26 participants, comprising the 25 participants who were included in the final analysis and the one participant who began using LDART but withdrew participation for a higher level of care.

Abbreviation: AUDIT, Alcohol Use Disorders Identification Test.

### 3.3 Acceptability

#### 3.3.1 Engagement

Participants accessed LDART on average 22.9 (SD = 4.3) out of 28 days, which was 81.8% of the days. There was no significant difference in the average number of days using LDART by week (*F*_3,54_ = 1.5, *p* = 0.2, *n* = 25; Figure S2). The average number of days participants used LDART was significantly greater than zero each week, including weeks where participants were not compensated for using LDART (Wilcoxon signed rank test *W* = 325.0, *p* < 0.0001). There was a non‐significant effect of participant compensation on engagement: average days using LDART on compensated weeks (i.e., weeks 1 and 3) were 11.9 (SD = 2.0) out of 14 days (85.0%), while average days using LDART on non‐compensated weeks (i.e., weeks 2 and 4) were 11.0 (SD = 2.7; 78.6% of the days; Wilcoxon matched‐pairs signed rank test sum of signed ranks *W* = −72, *p* = 0.06).

#### 3.3.2 Acceptability

Most participants (17/21, 81.8%) indicated that they would recommend LDART to someone who was trying to cut down or quit drinking (Table 2). Open‐ended responses confirmed that some found LDART to be helpful overall, describing LDART as “simple and uplifting” and a “creative” way to promote recovery.

**TABLE 2:** Acceptability ratings of LDART. Each statement was rated on a 5‐point scale, where 1 indicated “strongly disagree”, 3 indicated “neither agree nor disagree”, and 5 indicated “strongly agree”. Percentage of participants who rated a 4 (“agree”) or 5 (“strongly agree”) for each statement was calculated.

Statement	Rating, mean (SD)	Agree or strongly agree (%)
Setting goals each night was helpful for my recovery	4.4 (0.9)	85.7
Receiving video messages on days where I reached my goal was helpful for my recovery	4.4 (0.8)	81.8
Receiving written messages on days where I reached my goal was helpful for my recovery	4.3 (1.0)	85.7
Receiving video messages on days where I did not reach my goal was helpful for my recovery	4.1 (1.0)	81.8
Receiving written messages on days where I did not reach my goal was helpful for my recovery	4.0 (1.0)	72.7
The information on recovery resources in my community was helpful for my recovery	4.1 (1.0)	76.2
Logging on each night was too often	2.4 (1.1)	18.2
The time I spent using LDART each night was too long	1.7 (0.9)	9.1
I would recommend LDART to someone trying to cut down or quit drinking	4.2 (1.1)	81.0

*Note:* *n* = 21–22 participants (3 of the 25 participants who completed using LDART did not complete the acceptability ratings, and 1 participant did not answer all questions on the acceptability questionnaire).

##### 3.3.2.1 Feedback on Goal‐Setting

Most participants (18/21, 85.7%) found the goal‐setting component of LDART to be helpful (Table 2). Six participants mentioned in their interviews that goal‐setting made them feel responsible for and intentional about their recovery: “The experience of logging in each night was exciting for me because it was something that I had to do to be accountable… It made me think about what I wanted to do for the next day, and it helped me be more focused.” One participant appreciated reporting goal outcomes in a non‐binary manner: “What I loved about it was the target‐like thing. Because it wasn't necessarily [that] you have to be perfect. It's like, ‘Okay, I'm just gonna decide to be better tomorrow’.” Three participants mentioned that it would be helpful to set the goal in the mornings and report on the goals in the evenings (Table 3); however, four participants said that they preferred reporting on and setting goals in the evenings. Other suggestions for improvement included providing the option to set more personalized goals and to set multiple goals each night (Table 3).

**TABLE 3:** Suggestions for improvement from qualitative interviews.

LDART component	Suggestion for improvement (*n*)	Representative quote
Goal‐setting	Set goals in the morning and report at night (3)	“It's better to set the goals in the morning and then check‐in at night”
	Option to create more personalized goals (3)	“When they ask what are your predetermined goals – rather than ‘do something good for yourself’, maybe it's ‘do something good for people around you’”
	Option to set multiple goals (2)	“I wish I could set multiple goals. For example, one day, I wanted to be productive, but I also wanted to drink less”
Messages	Add subtitles to videos (2)	“It's hard to step away and listen to a video… For convenience, maybe including subtitles would be a good idea”
	Provide tips/strategies for not drinking (2)	“Having some bite‐sized information [about what drinking or not drinking could do to your body] that would be motivational and supportive”
	Remove videos that were not well‐received (2)	“Some were really good and genuine… there were maybe one or two videos where I just didn't feel the love”
Recovery organization information	Provide resource sheet of CROs (2)	“Maybe supply all the organizations' information again at the end, like a PDF”
	Provide more specific information (1)	“I got to do a little research on them, and be able to see if they… offer services in my area, or was it online?”
Others	Allow interactions between LDART participants (3)	“Expand the interaction or create interaction… It would be nice to create a group chat of people who are doing LDART”

Abbreviation: *n*, number of participants who expressed a similar sentiment in their interviews.

##### 3.3.2.2 Feedback on Messages

Most participants (72.7%–85.7%) found the messages from people associated with CROs to be helpful (Table 2). Participants wrote about feeling “the kindness and care” and said that it helped them feel like they were “not alone” on their recovery journey. Participants made comments such as, “It was nice having videos from people who understand how hard recovery is.” Six participants explicitly mentioned that they preferred videos over written messages as videos allowed for “a more personal connection.” One of those participants articulated the added value of video messages as compared to written messages: “It's not just the words. It's also the tone, it's something about facial expressions, the body movements, the environment they're in, whether it's a real background or not… This is what triggers all the senses and this helps us focus more on the message that's there.” Importantly, some noted that videos with people who seemed charismatic were more inspirational than videos where people seemed uncomfortable talking to a camera (Table 3).

Even though several participants preferred videos over written messages, some participants also enjoyed receiving the latter: “The texts were great – they feel more private because I'm just reading it in my own personal space. And I can kind of interpret it in a way I want to.” Another participant appreciated the convenience of written messages: “Maybe the text was more impactful because I like to read all of those, whereas [for videos] there were a couple of times I didn't pay enough attention because of where I was or didn't fully listen to the video.” This suggests that adding subtitles to the videos may improve accessibility (Table 3).

Two participants suggested providing informational support on how to reduce drinking in the messages (Table 3).

##### 3.3.2.3 Feedback on Recovery Resources

Most participants (16/21, 76.2%) found the information on recovery resources to be helpful (Table 2). In interviews, multiple participants mentioned not being familiar with these organizations prior to using LDART: “It was good to know that there's so many different spaces and places to go for people to get help, because a lot of people don't even know about these things because [those programs] are not broadcasted all the time.” Participants expressed a range of responses to receiving these resources, from not being interested (“I didn't look too much into that… I'm not really interested in going to meetings, personally”), to mild interest (“I took a look through all of them but not a deep dive into any of them specifically”), to actively exploring these resources (“I [chose] certain ones that I thought pertained really to me, [I made] notes of it”) and engaging with the CROs. Providing a resource sheet with information on all CROs was suggested to facilitate engagement with the CROs after learning about them through LDART (Table 3).

One participant mentioned appreciating the diversity of CROs available: “[I liked learning about] women [CROs] specifically, which is pretty cool. I feel like this drinking journey has been very men‐centric.” Another participant mentioned how identifying with the people in the videos influenced them to engage with the organization: “I checked out the [CRO] with the Muslim guy [in the video]. He is relatable to me because we're both Black.” This suggests the importance of providing a diversity of CROs and individuals who represent the CROs.

##### 3.3.2.4 Other Feedback

Participants provided other valuable feedback on how to improve the intervention (Table 3). Three participants mentioned finding ways to increase social interaction as part of the intervention, either by allowing interactions between LDART participants or having a member of the study team check in with participants each week.

### 3.4 Preliminary Efficacy

The percentage of past‐month drinking days was lower during (45.2 ± 34.6 days) and after (42.5 ± 34.2 days) using LDART than before using LDART (61.3 ± 31.2 days; Figure S3A). The percentage of past‐month heavy drinking days was also lower during (17.1 ± 22.5 days) and after (17.9 ± 23.2 days) using LDART as compared to before using LDART (27.1 ± 30.0 days; Figure S3B).

There was a striking increase in the average number of hours engaging with CROs from 2.4 ± 5.5 h before using LDART to 10.8 ± 25.4 h during LDART and 7.6 ± 12.7 h during the post‐intervention month. In terms of CRO engagement as a binary variable, 5/23 (21.7% of) participants had some engagement with CROs in the month before beginning LDART, compared to 9/23 (39.1% of) participants during LDART and 9/23 (39.1% of) participants in the month post‐intervention. Participants also engaged with a greater number of CROs during and after using LDART than before using LDART (Figure S3C). Participants collectively engaged with three CROs in the month before using LDART, versus with 11 CROs during and/or after LDART (Figure S3D). Among the 18 participants who had not engaged with CROs in the month prior to LDART, 5 of them (27.8%) engaged with at least one CRO during and/or after LDART. Among the five participants who had engaged with at least one CRO in the month prior to LDART, four of them (80.0%) engaged with at least one more CRO during and/or after LDART.

There was an increase in self‐efficacy over time, from 30.1 ± 4.7 before using LDART to 32.1 ± 5.0 after using LDART and 31.3 ± 5.3 at 1‐month post‐intervention. There was an increase in quality of life over time, from 55.1 ± 4.2 before LDART to 59.1 ± 4.2 at post‐intervention and 58.0 ± 4.6 at 1‐month post‐intervention. There was an increase in recovery capital over time, from 43.9 ± 8.0 before LDART to 46.9 ± 9.2 at post‐intervention and 45.3 ± 7.9 at 1‐month post‐intervention.

## 4 Discussion

This pilot study evaluated the feasibility, acceptability, and preliminary efficacy of LDART, a novel digital social support intervention designed to reduce hazardous alcohol use by increasing engagement with CROs. The study enrolled 30 adults who wanted to reduce their hazardous alcohol use to use LDART for a month. Study recruitment and retention rates suggest the feasibility of conducting this clinical trial. LDART acceptability was indicated by high rates of engagement with the intervention and high ratings of the helpfulness of LDART components. Outcome data suggest preliminary efficacy of LDART in reducing alcohol use and increasing engagement with CROs.

### 4.1 Feasibility and Acceptability of LDART

This study demonstrated the feasibility of its ability to recruit and retain participants. Recruitment of adults who engaged in past‐year hazardous alcohol use and who had some desire to cut down was feasible, at a rate of 10 participants per month, achieving our target sample size within a 3‐month period. Our high retention rate was comparable to retention rates used as benchmarks in other studies on digital interventions for reducing alcohol use, commonly 70%–80% (Gex et al. 2023; Merrill et al. 2024), which suggests that the study was not too burdensome for participants.

Engagement is a common challenge faced by mHealth app creators, as app engagement rarely continues after a few weeks (Baumel et al. 2019; Nwosu et al. 2022; Torous et al. 2025). Participants used LDART on average 81.8% of the nights for 4 weeks, which is comparable to acceptability benchmarks set in other interventional studies for alcohol involving daily survey completion with modest compensation (Merrill et al. 2024; Padovano et al. 2022). Since engagement did not significantly decrease across the 4 weeks, this suggests that extending LDART to be used over a longer duration may be plausible. This would be particularly relevant since addiction can be a chronic condition, and individuals may benefit from support over the entire course of early recovery (i.e., the first year), not merely the first month. Future qualitative studies will explore participants' preferred duration and frequency of using LDART.

Since we provided financial compensation for engaging with the intervention, we cannot say definitively that engagement would be similarly high without compensation, or that engagement would be similar between participants of differing financial situations. That said, our data indicate that the effect of compensating participants for using the intervention was modest and non‐significant. Compensation may also influence participants' responses (e.g., reporting the outcomes of their goals) and perceived helpfulness of the intervention, which we attempted to mitigate by informing participants that they were being compensated specifically for using the intervention, irrespective of what they self‐reported. Nonetheless, future trials of LDART for promoting recovery from hazardous alcohol use will not provide compensation for intervention engagement to gain a more accurate indication of acceptability and to disambiguate between intrinsic and extrinsic motivation.

Overall, participants reported that LDART was helpful for their recovery and would recommend it to someone who might need it. Of the components of LDART, participants reported appreciating most the goal‐setting feature. Digital interventions designed to facilitate daily goal‐setting in the context of problematic alcohol use often only allow setting goals specific to alcohol use (Merrill et al. 2024; Padovano et al. 2022). In line with recent shifts toward defining recovery in a holistic way beyond abstinence or cessation of heavy drinking (Hagman et al. 2022), LDART was designed for goal‐setting of recovery‐related goals, not strictly alcohol‐related goals. Based on participant feedback from qualitative interviews, future iterations of LDART may allow participants to set multiple goals and have them report on whether they felt they were successful overall (and would thus receive a congratulatory goal‐reached video) or not (and receive an encouraging goal‐missed video), or create additional videos for partial successes. We may also provide participants with the option of setting their goals in the morning and reporting on the outcome at night.

The pre‐recorded videos from peers were well‐received by participants. That said, some participants noted that some videos were more effective than others. Future studies should identify specific verbal and nonverbal properties in videos that might be associated with being helpful for people in recovery; this information can then be used to identify and remove videos that may not be as effective to optimize the intervention. Additionally, in response to participants mentioning the value of including tips or strategies on how to reduce alcohol use, future messages could include more informational support in addition to emotional support.

Another key feature of LDART is the provision of CRO information. Our baseline assessment and qualitative data revealed that most participants were unaware of CROs beyond 12‐step programs. This has been reported in other studies (Beck et al. 2025; Kelly et al. 2017). This affirms the value of LDART in increasing awareness of these CROs. Our results indicated that participants were more likely to engage with non‐12‐step CROs during and after using LDART than before using LDART, suggesting that LDART was useful not only in providing information on CROs but also in encouraging participants to engage with the CROs. In response to participant feedback, the next version of LDART may include a feature that allows participants to send the CRO information they see on LDART to themselves via text or email so they have a record of the information to further facilitate engagement with the CROs.

### 4.2 Preliminary Efficacy and Potential Mechanisms of Change

The decrease in self‐reported alcohol consumption during this study suggests that LDART may be a promising intervention to facilitate reduced alcohol use. However, a larger sample size in a randomized design with a control group is needed to determine the extent to which these decreases in alcohol consumption are attributable to LDART. Additionally, due to the remote format of this study, alcohol use was measured entirely by self‐report without biochemical verification of abstinence.

The increases in self‐efficacy and CRO engagement (i.e., a proxy for improvements in socio‐structural factors) observed indicate potential SCT‐based mechanisms through which LDART may exert therapeutic effects. This aligns with a recent systematic review, which identified self‐efficacy and social support as variables with the strongest empirical support as mediators of alcohol and other drug treatment (Magill et al. 2023, 2025; Maisto et al. 2024). Based on the small sample size and lack of control condition, these preliminary measures are exploratory and limited, and we cannot form any conclusions regarding the mechanisms of LDART. A larger trial with a randomized design facilitating mediation analyses, possibly incorporating time‐linked proximal measures (e.g., weekly or ecological momentary assessments), is thus needed.

### 4.3 Limitations

In addition to the limitations mentioned above regarding compensating participants for using the intervention and the small sample size, other limitations exist. First, our eligibility criteria included a self‐reported desire to cut down or quit drinking, which may have created a selection bias toward people who had relatively high levels of motivation to reduce their alcohol use. This is reflective of real‐world situations in which individuals are unlikely to seek out and use digital interventions for reducing alcohol use (e.g., mHealth apps in app stores) without some inherent motivation to reduce their drinking. Nonetheless, we intentionally made this criterion broad (“Do you have some desire to cut down or quit drinking?”—Yes/No) to maximize generalizability across a range of motivational levels, and will likely preserve this criterion in subsequent trials. Second, the study approach involved an interactive onboarding procedure over Zoom, where a staff member walked participants through LDART by screensharing, which may have inflated engagement levels more than if participants had not been guided through the process. Future studies could incorporate a more hands‐off approach to mimic real‐world situations in which participants find and use digital interventions themselves. Third, our sample was predominantly White and geographically constrained to Connecticut, USA; future studies should increase diversity to determine generalizability and to identify whether LDART is particularly effective/ineffective for participants with specific demographic, geographical, and clinical characteristics. Fourth, the perspectives of the participants who were randomly selected to participate in the qualitative interview may not generalize to the larger sample of participants. However, any differences between the two groups of participants did not appear to impact their frequency of use or overall ratings of LDART. Additionally, the larger sample of participants who did not participate in the interview had the option to write in their thoughts about LDART in the acceptability questionnaire, so they had the opportunity to share their perspectives. Future studies should ensure a more representative sample of participants selected for interviews. Fifth, our measure of self‐efficacy was not specific to alcohol use and thus may not be sensitive to participants' alcohol‐specific mastery experience. Although it is possible for some interventions to have a generalized clinical impact on self‐efficacy (Magill et al. 2025), our future studies will include both domain‐specific measures of self‐efficacy (DiClemente et al. 1994) and self‐efficacy more generally.

### 4.4 Conclusions

This pilot study suggests LDART is feasible and acceptable as a digital intervention for adults who are interested in reducing hazardous alcohol use. Outcome data suggest that LDART may be a useful tool for reducing hazardous alcohol use and increasing CRO engagement. These promising results warrant testing LDART in future studies such as a randomized controlled trial. This study contributes to the emerging body of research investigating SCT‐informed interventions for problematic alcohol use.

## Author Contributions

L.Y.M.: conceptualization, funding acquisition, methodology, formal analysis, writing – original draft, writing – review and editing. T.J.C., A.M.S., and C.N.: formal analysis. T.L.S.: writing – original draft. S.K.‐S., G.K., B.D.K., and M.N.P.: supervision, writing – review and editing.

## Funding

This study is funded by an Early Career Psychologist research grant from the Society of Addiction Psychology (Division 50, American Psychological Association) to L.Y.M. L.Y.M. was additionally supported by T32‐funded postdoctoral training fellowships (T32DA019426 and T32DA022975) and is supported by a career development award (K12DA000167) from the National Institute on Drug Abuse.

## Conflicts of Interest

The authors declare that they have no known competing financial interests or personal relationships that could have appeared to influence the work reported in this paper. B.D.K. is a paid consultant to CBT4CBT LLC, which makes CBT4CBT available to qualified clinical providers and organizations on a commercial basis. The conflict is managed through Yale University. M.N.P. discloses that he has consulted for and advised Baria‐Tek and Boehringer Ingelheim; been involved in a patent application with Yale University and Novartis; received research support from the Mohegan Sun Casino and the Connecticut Council on Problem Gambling; consulted for or advised legal, non‐profit, healthcare and gambling entities on issues related to impulse control, internet use and addictive behaviors; performed grant reviews; edited journals/journal sections; given academic lectures in grand rounds, CME events, and other clinical/scientific venues; and generated books or chapters for publishers of mental health texts.

## Supporting information


**Table S1:** List of CROs featured in LDART.
**Table S2:** Comparison of demographic information between participants who were versus were not selected to participate in the qualitative interview.
**Table S3:** Comparison of acceptability ratings of LDART between participants who were and were not selected to participate in the qualitative interview.
**Figure S1:** Mapping of LDART intervention components onto SCT constructs.
**Figure S2:** Number of days participants used LDART, by week.
**Figure S3:** Preliminary efficacy data.

## Data Availability

De‐identified data from this study are not available in a public archive but can be made available for reasonable requests by emailing the corresponding author.
