# Supplementary material for: Development and Pilot Test of a Novel Digital Social Support Intervention for Reducing Hazardous Alcohol Use
Source: Alcohol Clin Exp Res (Hoboken). 2026 Apr 23;50:e70301. doi: 10.1111/acer.70301 (PMC13106737; doi:10.1111/acer.70301)
Supplement: Supplementary file 1 — Table S1: List of CROs featured in LDART. Table S2: Comparison of demographic information between participants who were versus were not selected to participate in the qualitative interview. Table S3: Comparison of acceptability ratings of LDART between participants who were and were not selected to participate in the qualitative interview. Figure S1: Mapping of LDART intervention components onto SCT constructs. Figure S2: Number of days participants used LDART, by week. Figure S3: Preliminary efficacy data. [file ACER-50-0-s001.docx]

**Development and pilot test of a novel social support intervention for reducing hazardous alcohol use**

Supplementary Figures & Tables:

Supplementary Table S1: List of CROs featured in LDART

Supplementary Figure S1: Mapping of LDART intervention components onto SCT constructs

Supplementary Table S2: Comparison of demographic information between participants who were versus were not selected to participate in the qualitative interview

Supplementary Figure S2: Number of days participants used LDART, by week

Supplementary Table S3: Comparison of acceptability ratings of LDART between participants who were and were not selected to participate in the qualitative interview.

Supplementary Figure S3: Preliminary efficacy data

Supplementary Methods

Supplementary Results

Supplementary References

**Supplementary Table S1**

| CRO name | Organization type | Number of videos |
| --- | --- | --- |
| LifeRing Secular Recovery | Mutual help organization | 1 |
| Millati Islami | Mutual help organization | 2 |
| Moderation Management (MM) | Mutual help organization | 2 |
| SMART Recovery | Mutual help organization | 3 |
| Women for Sobriety (WFS) | Mutual help organization | 1 |
|  |  |  |
| Connecticut Community for Addiction Recovery (CCAR) | Recovery community center | 4 |
|  |  |  |
| Busy Living Sober | Recovery podcast | 1 |
| One Day at a Time | Recovery podcast | 1 |
| The Way Out | Recovery podcast | 2 |
|  |  |  |
| The Phoenix | Sober active community | 1 |
|  |  |  |
| Alcoholics Anonymous (AA) | Mutual help organization | 0 |
| Recovery Dharma | Mutual help organization | 0 |
| Recovery International | Mutual help organization | 0 |
| Refuge Recovery | Mutual help organization | 0 |
| Secular Organization for Sobriety | Mutual help organization | 0 |
| SHE RECOVERS | Mutual help organization | 0 |
| Wellbriety | Mutual help organization | 0 |
| Young People in Recovery | Mutual help organization | 0 |
|  |  |  |
| Musical Intervention | Sober creative-collaborative space | 0 |

**Supplementary Table S1: List of community-based recovery organizations (CROs) featured in LDART.** CROs that contributed videos had their information displayed after a video message of someone from their organization was played. CROs that did not contribute videos had their information displayed after a written message (generated as a composite of transcripts of the video messages) was shown. *n* = 19 CROs, comprising 10 CROs that contributed 18 videos and 9 CROs that did not contribute videos. The final (28th) video was of the first author.

**Supplementary Figure S1**

**
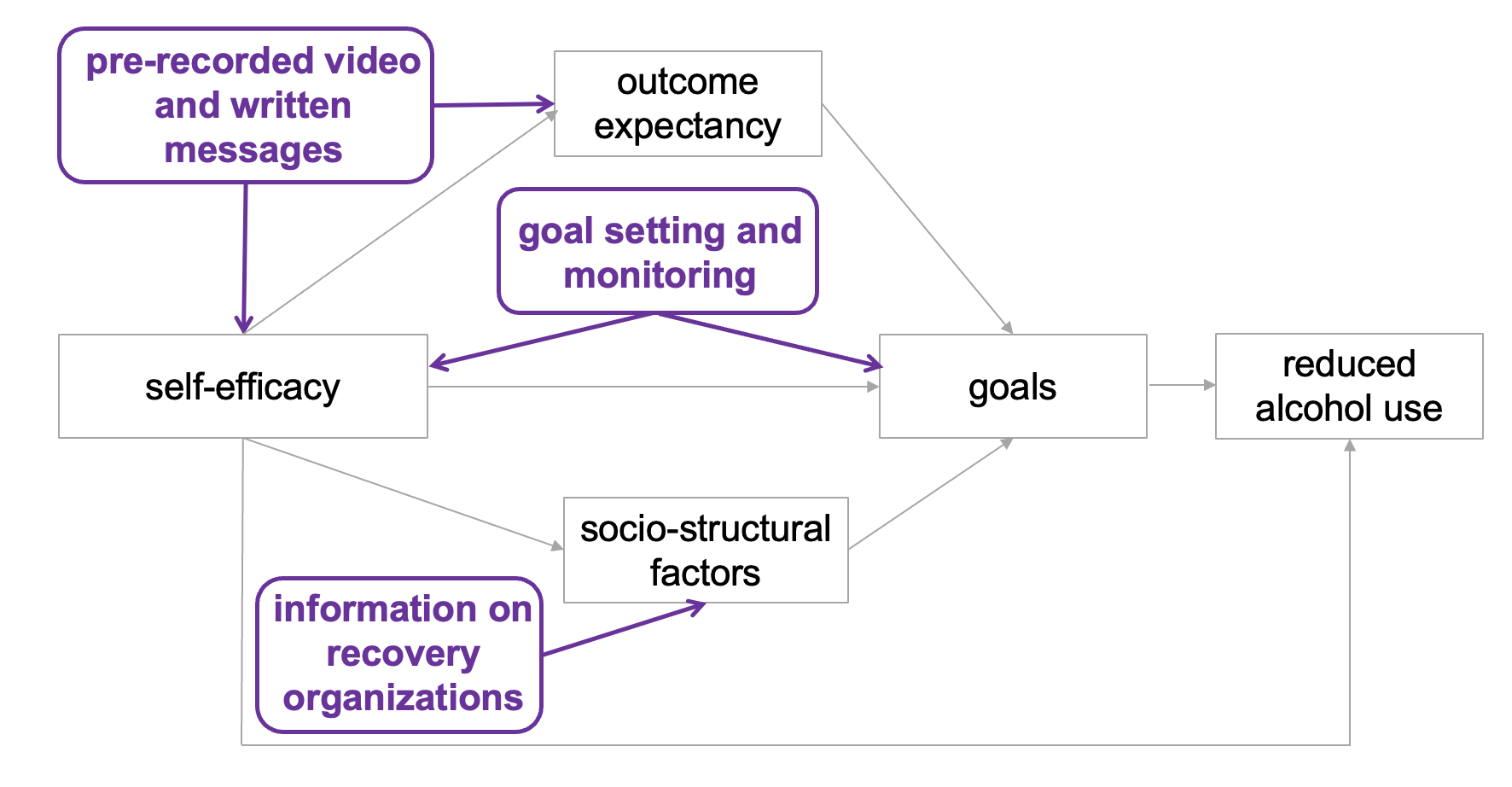
**

**Supplementary Figure S1: Mapping of LDART intervention components onto social cognitive theory (SCT) constructs.** Thin grey boxes and arrows indicate relationships between SCT constructs as depicted in Bandura (2004). Thick purple rounded boxes and arrows indicate LDART’s therapeutic components and ways in which they are thought to impact SCT constructs.

**Supplementary Table S2**

|  | **QI participants (*n*=10)** | **Non-QI participants (*n*=16)** | **Test statistic** | ***p*-value** |
| --- | --- | --- | --- | --- |
| ***Demographic measures*** |  |  |  |  |
| **Age in years, mean (SD)** | 40.7 (10.3) | 46.9 (10.3) | *t*=1.5 | 0.2 |
| **Gender, *n* (%)** |  |  |  |  |
| Female | 3 (30.0) | 11 (68.8) | χ^2^=3.7 | 0.05 |
| Male | 7 (70.0) | 5 (31.3) |  |  |
| **Race, *n* (%)** |  |  |  |  |
| American Indian / Alaskan Native | 0 (0.0) | 1 (6.3) | χ^2^=0.2 | 0.6 |
| Black / African-American | 2 (20.0) | 4 (25.0) |  |  |
| White / European-American | 8 (80.0) | 10 (62.5) |  |  |
| Prefer not to answer | 0 (0.0) | 1 (6.3) |  |  |
| **Ethnicity, *n* (%)** |  |  |  |  |
| Hispanic / Latinx | 1 (10.0) | 1 (6.3) | χ^2^=0.1 | 0.7 |
| Not Hispanic / Latinx | 9 (90.0) | 15 (93.8) |  |  |
| **Education, *n* (%)** |  |  |  |  |
| High school or less | 0 (0.0) | 2 (12.5) | χ^2^=1.7 | 0.2 |
| Some college | 3 (30.0) | 7 (43.8) |  |  |
| Associate’s, Bachelor’s or above | 7 (70.0) | 7 (43.8) |  |  |
| **Income, *n* (%)** |  |  |  |  |
| Less than $50,000 | 5 (50.0) | 7 (43.8) | χ^2^=1.7 | 0.4 |
| $50,000 - $99,999 | 4 (40.0) | 4 (25.0) |  |  |
| More than $100,000 | 1 (10.0) | 5 (31.2) |  |  |
| **Employment, *n* (%)** |  |  |  |  |
| Working full-time | 4 (40.0) | 8 (50.0) | χ^2^=2.0 | 0.6 |
| Working part-time | 1 (10.0) | 3 (18.8) |  |  |
| Stay-at-home parent | 0 (0.0) | 1 (6.3) |  |  |
| Retired / Unemployed | 3 (30.0) | 3 (18.8) |  |  |
| Other / Prefer not to answer | 2 (20.0) | 1 (6.3) |  |  |
| **Access to devices, *n* (%)** |  |  |  |  |
| Android phone | 4 (40.0) | 8 (50.0) | χ^2^=4.6 | 0.2 |
| iPhone | 7 (70.0) | 8 (50.0) |  |  |
| iPad | 4 (40.0) | 1 (6.3) |  |  |
| Laptop/Computer | 5 (50.0) | 12 (75.0) |  |  |
| ***Alcohol measures,* mean (SD)** |  |  |  |  |
| Years of problematic alcohol use | 15.3 (14.0) | 11.2 (9.5) | *U*=72 | 0.7 |
| Age began regular alcohol use | 17.7 (2.5) | 26.3 (13.6) | *U*=38 | 0.03 |
| Past-year AUDIT score | 23.5 (9.9) | 22.3 (8.2) | *t*=0.4 | 0.7 |
| Past-month drinking days | 13.2 (8.1) | 20.2 (8.6) | *U*=46 | 0.07 |
| Past-month heavy drinking days | 6.9 (7.6) | 9.6 (9.7) | *U*=71 | 0.6 |

**Supplementary Table S2: Comparison of demographic information between participants who were versus were not selected to participate in the qualitative interview (QI).** Demographic information on the full sample (*n*=26) is in Table 1. AUDIT: Alcohol Use Disorders Identification Test. To handle instances where χ^2^ test could not be conducted due to the presence of zero values, the following modifications were performed: for Race, only the proportion of Black and White participants between the two groups was compared; for Education, “high school or less” was combined with “some college”; for Employment, “stay-at-home parent” was combined with “working full-time”.

**Supplementary Figure S2**

**
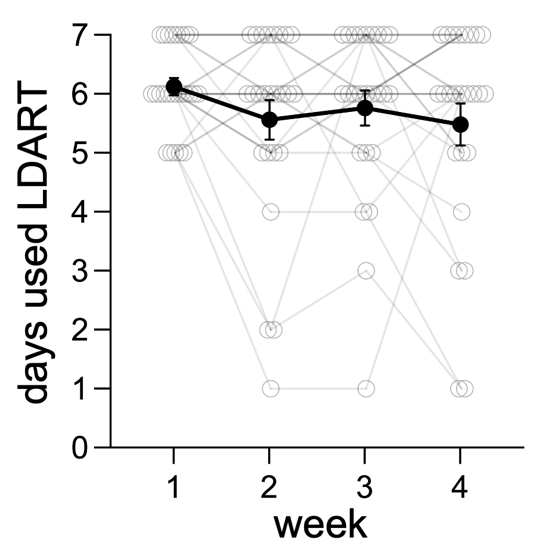
**

**Supplementary Figure S2: Number of days participants used LDART, by week.** *n* = 25 participants. Thick black line with solid black circles indicates the average; thin grey lines with open grey circles indicate individual participant data. There was no significant difference in the average number of days using LDART by week (*F*_3,54_=1.5, *p*=0.2).

**Supplementary Table S3**

| **Statement** | **QI participants mean rating (SD)** | **Non-QI participants mean rating (SD)** | ***t*** | ***p*** |
| --- | --- | --- | --- | --- |
| *Setting goals each night was helpful for my recovery.* | 4.8 (0.4) | 4.1 (0.9) | 1.9 | 0.07 |
| *Receiving video messages on days where I reached my goal was helpful for my recovery.* | 4.1 (0.8) | 4.5 (0.8) | 1.1 | 0.3 |
| *Receiving written messages on days where I reached my goal was helpful for my recovery.* | 4.5 (0.8) | 4.1 (1.1) | 0.8 | 0.5 |
| *Receiving video messages on days where I did not reach my goal was helpful for my recovery.* | 4.3 (0.9) | 4.1 (1.1) | 0.4 | 0.7 |
| *Receiving written messages on days where I did not reach my goal was helpful for my recovery.* | 4.1 (1.0) | 3.9 (1.1) | 0.4 | 0.7 |
| *The information on recovery resources in my community was helpful for my recovery.* | 4.1 (0.9) | 4.1 (1.2) | 0 | 0.9 |
|  |  |  |  |  |
| *Logging on each night was too often.* | 2.8 (1.2) | 2.2 (1.0) | 1.2 | 0.3 |
| *The time I spent using LDART each night was too long.* | 1.6 (1.1) | 1.8 (0.8) | 0.4 | 0.7 |
|  |  |  |  |  |
| *I would recommend LDART to someone trying to cut down or quit drinking.* | 4.0 (1.6) | 4.4 (0.8) | 0.7 | 0.5 |

**Supplementary Table S3: Comparison of acceptability ratings of LDART between participants who were and were not selected to participate in the qualitative interview (QI).** Each statement was rated on a 5-point scale, where 1 indicated “strongly disagree”, 3 indicated “neither agree nor disagree”, and 5 indicated “strongly agree”. Percentage of participants who rated a 4 (“agree”) or 5 (“strongly agree”) for each statement was calculated. *n* = 8 participants in the QI group and *n* = 14 participants in the non-QI group. There was also no difference in average number of days QI participants used LDART (22.5+-5.1) versus non-QI participants (23.6+-2.7), *t*_23_=0.64, *p*=0.53.

**Supplementary Figure S3**

**
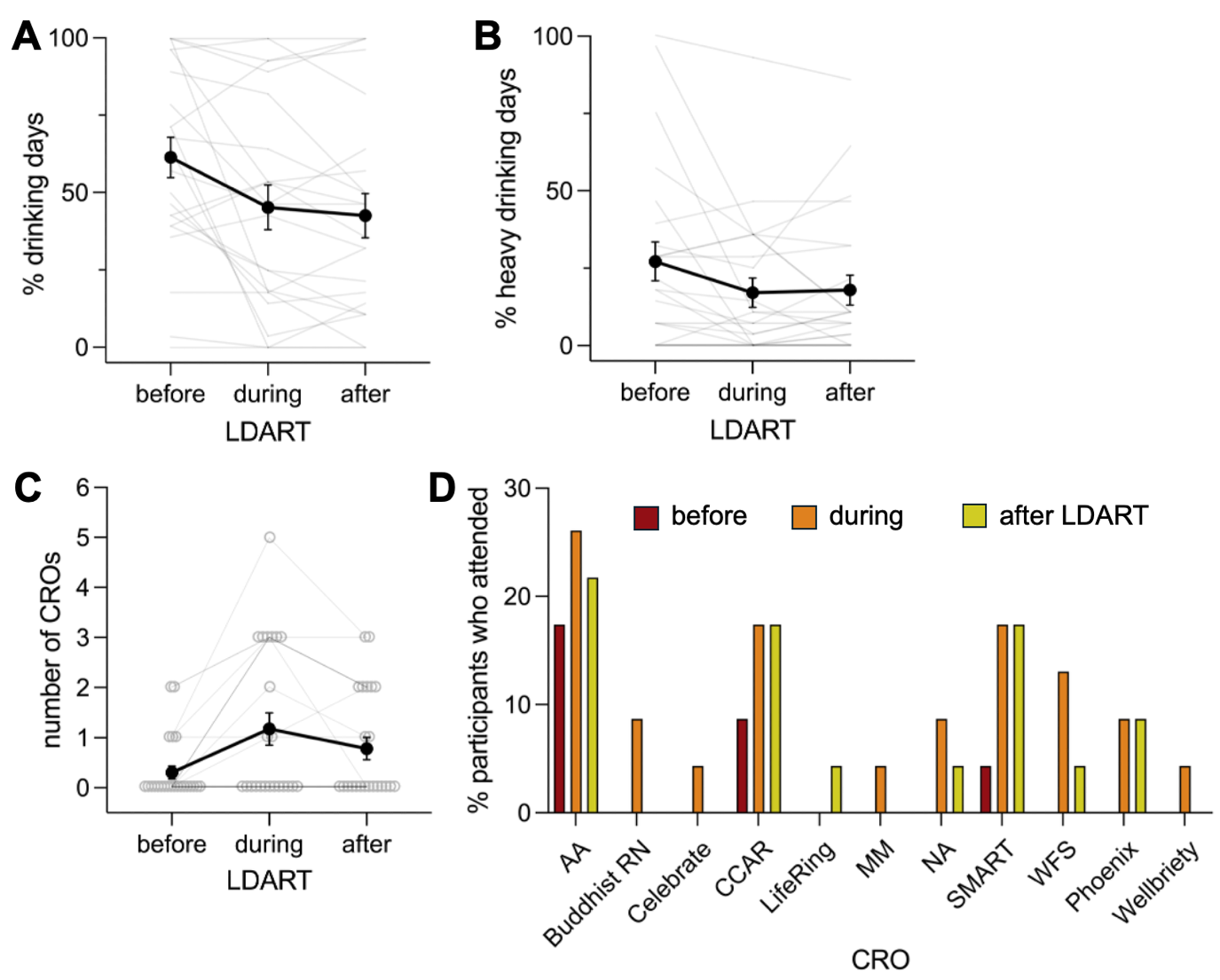
**

**Supplementary Figure S3: Preliminary efficacy data**. Average percentage of **A)** drinking days, **B)** heavy drinking days, and **C)** number of CROs attended, calculated for three time windows: the 28 days prior to beginning using LDART (“before”), the 28 days that participants used LDART (“during”), and the 28 days after participants completed using LDART (“after”). *n* = 23 participants. (2 of the 25 participants did not have data at their third time point so were excluded from analyses.) Thick black line with solid black circles indicates the average; thin grey lines with open grey circles indicate individual participant data. Error bars indicate standard error of the mean. **D)** Percentage of participants who engaged with each CRO, before (maroon), during (orange), and after (yellow) using LDART. CRO acronyms are explained in Supplementary Table S1.

**Supplementary Methods**

**Conceptual framework of LDART**

LDART is grounded in social cognitive theory (SCT). SCT has four constructs associated with behavior change: *goal setting* (i.e., setting specific and achievable objectives for behavior change), *self-efficacy* (i.e., the belief in one’s ability to change one’s behavior), *outcome expectations* (i.e., the anticipated consequences of engaging in the behavior), and *sociocultural factors* (i.e., environmental influences that may facilitate or hinder behavior change) (Bandura, 2004). Few psychosocial interventions explicitly target all four SCT constructs to facilitate behavior change, which may have synergistic therapeutic effects. Indeed, some have noted that “integrating social influence, peer support with education materials, and digital technology-based initiatives [can create] notable guiding principles for preventative, promotive, and curative health behaviors among target populations worldwide” (Islam et al., 2023). An overview of the theoretical framework linking specific LDART components with SCT constructs is provided in Supplementary Figure S1. Details about each component of the intervention and its theoretical justification are presented here.

***Goal setting and monitoring***

*Goal setting* aligns with the agentic perspective of SCT, which conceptualizes goals as directly linked to behavior change (Bandura, 2004). Goal setting has also been acknowledged by other theories (e.g., goal setting theory) as an important component of health behavioral change (Epton et al., 2017; Locke & Latham, 2002; Strecher et al., 1995).

We opted to implement goal setting on a daily basis, as have other interventions for reducing alcohol use (Adamson et al., 2010; Padovano et al., 2022), as opposed to longer time frames such as weekly or monthly intervals. This was due to several reasons: (1) Daily goals are easier to achieve than longer-term goals (e.g., staying sober for 24 hours is easier than staying sober for 168 hours). This increases the likelihood of reaching one’s goals, which may increase self-efficacy through personal mastery; (2) Daily goals may feel more manageable than longer-term goals. This decreases the perceived goal difficulty, which can increase motivation for pursuing one’s goals (Etkin, 2019). This also aligns with the common adage in recovery spaces to take things “one day at a time” when pursuing sobriety; (3) Daily goals (as opposed to longer-term goals) may subtly reframe how “success” is defined in recovery and make “failures” feel less demoralizing. For example, someone whose goal is to stay sober for a month who drank on one day might traditionally view this as a “failure,” whereas daily goal-setting may encourage a more positive outlook: “I succeeded in staying sober on 29 out of 30 days.” This moves away from the traditional conceptualization of “relapse” as a negative endpoint or outcome, and towards a SCT conceptualization that frames slips as a “transitional process” (Larimer et al., 1999); (4) Daily goal-setting allows for daily reinforcement for pursuing one’s goals (via the motivational messages) instead of waiting 7 days before receiving reinforcement. This is particularly relevant for individuals with AUD, who tend to discount future rewards more steeply than individuals without AUD (Petry, 2001).

Each night, participants selected a recovery goal to work towards the next day, from a list comprising seven pre-set goals and up to three personalized goals written by the participant. Some pre-set goals were directly related to alcohol use (e.g., “not drink”, “drink less than usual”), and others were related to improving social support, such as “go to a support group meeting”, “go to a recovery community center”, and “spend time with someone supportive”. Repeated failure to achieve goals can reduce self-efficacy (Ordóñez et al., 2009; Pearson, 2012), so we included some pre-set goals that were relatively easy to achieve, such as, “do something related to self-care” and “make it through the day”, to prevent participants from setting goals that felt unachievable. Since setting personally relevant goals is associated with greater self-efficacy as compared to when assigned goals (Lozano & Stephens, 2010; Magill et al., 2022), participants could also write in up to three personalized goals prior to beginning LDART.

Goal outcomes are generally conceptualized as a dichotomous outcome (i.e., goal reached versus goal missed), which fails to consider the effort put into reaching the goal or improvements in skills or behavior. In LDART, participants indicated how close they were to reaching their goal by clicking on a dartboard. For example, they clicked inside the bullseye (red region) if they reached their goal, in the orange region next to the bullseye if they almost reached their goal, or in the grey region most distant from the bullseye if they were not close to reaching their goal (Figure 1). This allowed participants to report their progress toward their goals in a way that acknowledges their effort and progress towards reaching goals.

Bandura (1991) and others theorized that individuals compare their past behavior with current behavior to evaluate progress and inform future goals (Lewis et al., 2019). This self-referential function allows individuals to set more ambitious goals if they regularly reach their goals, or to set more realistic goals that are more achievable if they are not reaching their goals. After each week (i.e., on days 7, 14, 21, and 28), LDART displayed the percentage of goals each participant reached alongside a brief written message either congratulating participants if they reached 50% or more of their goals that week, or a brief encouraging message if they reached less than 50% of their goals that week. Highlighting one’s success rate aligns with the SCT factor of having previous experiences of *personal mastery* (Bandura, 1977), by bringing to mind one’s successes in recovery, no matter how big or small their daily recovery goals were.

Overall, the nightly goal setting, reporting, and monitoring component of LDART was designed to facilitate the pursuit of goals, which is directly linked to behavior change based on SCT (Supplementary Figure S1). Additionally, we posit that *self-efficacy* may be increased by gaining a sense of *personal mastery* from recognizing one’s *performance accomplishments*, which is both directly and indirectly linked to behavior change based on SCT (Supplementary Figure S1).

***Video and written messages***

Details on video collection and characterization will be available in a companion manuscript. Briefly, individuals associated with CROs were invited to contribute videos for LDART between August and December 2023. They accessed a Qualtrics survey (<https://yalesurvey.ca1.qualtrics.com/jfe/form/SV_9yn3RYx8GdgMOWi>) where they were introduced to LDART and given instructions for creating two videos: a celebratory video to be shown on days when the participant reaches their goal, and an encouraging video to be shown on days when the participant does not reach their goal. The videos were recorded on their own devices and uploaded to Qualtrics. They then provided information on their affiliated recovery resource and were compensated with a $40 electronic gift card.

Eighteen individuals associated with CROs contributed videos, and the 19^th^ video came from the first author and was shown on the last night of the intervention. Because only 19 videos were obtained for the 28 intervention days, the remaining 9 days displayed written messages instead of video messages. These written messages were compilations of transcripts from multiple individuals’ videos and were designed to have approximately the same number of words as the video messages.

Seeing others succeed at behavior change may increase self-efficacy through *vicarious experience*, as the viewers come to believe that they too can be successful in their efforts (Bandura, 1977). We thus believe that receiving messages from individuals who have successfully overcome addiction can increase self-efficacy in LDART participants (Supplementary Figure S1). Additionally, these messages provide *verbal persuasion* (i.e., social persuasion that one possesses the capabilities to master difficult situations), which is another source of self-efficacy (Bandura, 1977). These messages are also thought to influence *outcome expectancies* (Bandura, 1977). Participants using LDART learn that reaching goals is associated with receiving congratulatory messages and missing goals is associated with receiving encouraging, empathetic messages; in either case, *efforts* to reach recovery goals are met with positive messages (i.e., positive outcome expectancies), which could motivate individuals to keep trying to reach their goals (Supplementary Figure S1). This is in contrast to situations where individuals may have developed negative outcome expectancies in response to trying to reach their recovery goals, for example if their successes are minimized or if they feel judged for “failing” to reach their goal.

***Information on recovery organizations***

*Socio-structural factors* in a person’s environment may facilitate or impede behavior change (Bandura, 2004). Even though several CROs exist as freely and publicly available online and/or in-person resources, many people are not familiar with them and thus do not access these sources of social support that may facilitate recovery. In LDART, we address this socio-structural barrier by providing information on 19 CROs to increase awareness of these CROs. A full list of the 19 CROs featured in LDART is provided in Supplementary Table S1. Here we use the term “CROs” to broadly encompass non-clinical, community-based, organizations that provide free peer support services from individuals in recovery from addiction. This mostly comprised mutual-help groups (e.g., Moderation Management, SMART Recovery, Women for Sobriety). Individuals associated with sober active communities (e.g., The Phoenix) and hosts of addiction recovery podcasts were also invited, consistent with the acknowledgement by the Substance Abuse and Mental Health Services Administration (SAMHSA) that “social and recreational recovery infrastructures and social media” can be important for sustaining recovery (SAMHSA, 2016). Additionally, by showing messages from individuals who are associated with the CROs prior to providing information on the CRO, the goal was to provide “a face and a voice” to each CRO and make CROs feel more relatable, with the goal of reducing psychological barriers to engaging with them.

***Integrating the three components***

These three therapeutic components were incorporated into a web-based intervention created on Qualtrics. Each participant received a unique Qualtrics link to access each night for 28 nights, which allowed personalization to each participant (e.g., participant’s first name and goal that was set would be displayed each night). It had built-in functionalities in place such that the intervention would not advance unless the participant accesses the link on the correct date, to prevent participants from prematurely advancing through the survey. To facilitate participation, participants could choose to receive nightly email and/or text notifications to access the website, at a time of their choice.

Video, text, and CRO information content were assembled in a manner to keep things approximately even between weeks. For example, there was a similar distribution of videos of women versus men, videos versus written messages, and secular versus spiritual/religious CROs across weeks. The video messages, written messages, and CRO information were displayed in the same order to each participant.

**Transparency and openness**

The study protocol was published prior to recruitment (McCurdy et al., 2023). Almost all study components were identical to those reported in the study protocol, with these following differences: 1) The Stages of Change Readiness and Treatment Eagerness Scale (Miller & Tonigan, 1996) instrument was not used as an exclusion criteria to measure “desire to cut down or quit drinking”; this was done via self-report on a single item instead to reduce participant burden; 2) the Drug Abuse Screening Test (Skinner, 1982) was not administered at baseline to reduce participant burden; 3) the Multidimensional Scale of Perceived Social Support (Zimet et al., 1990) was not administered at baseline as it did not capture social support regarding CROs; 4) hours spent engaging with CROs was not collected via Timeline Follow-back; participants were instead asked to estimate how many hours they spent at each CRO within the past 28-day window, to reduce participant burden; 5) the binary version of CRO engagement and number of CROs engaged are variables that were not originally listed as secondary outcomes; 6) the 10-item brief assessment of recovery capital (BARC) questionnaire was used instead of the 50-item assessment of recovery capital questionnaire (Groshkova et al., 2013) to reduce participant burden; and 7) the general self-efficacy scale (Schwarzer et al., 1995) was added as an exploratory variable.

This study was registered on ClinicalTrials.gov ([NCT06022107](https://clinicaltrials.gov/study/NCT06022107)) prior to recruitment. All components of the study are identical to those reported on ClinicalTrials.gov, with the addition of #5 and #7 mentioned above.

**Supplementary Results**

Similar results were obtained when excluding the 6 participants who self-reported having at least one heavy drinking day in the past month, but then did not actually endorse any heavy drinking days on the Timeline Follow-back the night before beginning LDART.

***Participant characteristics***

In general, participants were mostly non-Hispanic White adults in their 40s, with a near equal number of women and men. Eighty percent of participants had a past-year AUDIT score of 15 or more, indicating a likelihood of moderate-to-severe alcohol use disorder. Participants had 19.2 (SD=8.0) drinking days and 11.2 (SD=8.6) heavy drinking days on average in the past month. At baseline, participants were familiar with 2 CROs on average, with most participants (12/19, 63.2%) having only heard of 12-step programs (i.e., Alcoholics Anonymous and/or Narcotics Anonymous). Approximately half of the participants (10/19, 52.6%) had never attended a CRO meeting/event in their lifetime.

***Acceptability***

Participants accessed LDART on average 22.1 (SD=4.5) out of 28 days, which was 78.9% of the days. Although there was a decrease in average days using LDART across the weeks (6.0 days during week 1 versus 5.2 days during week 4), there was no significant difference in the average number of days using LDART by week (*F*_3,54_=1.2, *p*=0.3, *n*=19). There was a non-significant effect of participant compensation on engagement: average days using LDART on compensated weeks (i.e., weeks 1 and 3) was 11.6 (SD=2.1) out of 14 days (82.9%), while average days using LDART on non-compensated weeks (i.e., weeks 2 and 4) was 10.6 (SD=2.8; 75.7% of the days; *t*_18_=1.9, *p*=0.07, *n*=19).

***Preliminary efficacy***

The number of past-month drinking days was lower during (14.3±8.8 days) and after (13.2±9.4 days) using LDART than before using LDART (19.1±7.6 days). The number of past-month heavy drinking days was also significantly lower during (6.4±6.7 days) and after (6.5±7.1 days) using LDART as compared to before using LDART (10.3±8.2 days). There was a striking increase in average number of hours engaging with CROs from 1.5±4.1 hours before using LDART to 12.4±29.2 hours during LDART and 5.7±9.3 hours during the post-intervention month. In terms of CRO engagement as a binary variable, 3/17 (17.6% of) participants had some engagement with CROs in the month before beginning LDART, compared to 6/17 (35.3% of) participants during LDART and in the month post-intervention.

There was an increase in number of CROs with which participants engaged over time. In the month before using LDART, participants collectively engaged with 3 CROs (Alcoholics Anonymous, CCAR, SMART Recovery). During LDART, they collectively engaged with 8 CROs (the same 3 CROs as before, plus Buddhist Recovery Network, Celebrate Recovery, Narcotics Anonymous, Women for Sobriety, and The Phoenix), and 6 CROs during the post-intervention month. Among the 14 participants who had not engaged with CROs in the month prior to LDART, 3 of them (21.4%) engaged with at least one CROs during and/or after LDART. Among the 3 participants who had engaged with at least one CRO in the month prior to LDART, all 3 of them (100%) went to at least one more CRO during and/or after using LDART.

There was an increase in self-efficacy over time, from 29.6±4.4 before LDART to 32.3±4.6 after using LDART and 31.5±4.7 at one-month post-intervention. There was an increase in quality of life over time, from 54.0±5.1 before LDART to 59.7±5.2 at post-intervention and 60.2±5.9 at one-month post-intervention. There was an increase in recovery capital over time, from 43.0±7.6 before LDART to 47.1±9.5 at post-intervention and 45.7±8.4 at one-month post-intervention.

**Supplementary References**

Adamson, S. J., Heather, N., Morton, V., & Raistrick, D. (2010). Initial preference for drinking goal in the treatment of alcohol problems: II. Treatment outcomes. *Alcohol Alcohol*, *45*(2), 136-142. <https://doi.org/10.1093/alcalc/agq005>

Bandura, A. (1977). Self-efficacy: Toward a unifying theory of behavioral change. *Psychological Review*, *84*(2), 191-215. <https://doi.org/10.1037/0033-295X.84.2.191>

Bandura, A. (1991). Social cognitive theory of self-regulation. *Organizational Behavior and Human Decision Processes*, *50*(2), 248-287. <https://doi.org/https://doi.org/10.1016/0749-5978(91)90022-L>

Bandura, A. (2004). Health promotion by social cognitive means. *Health Educ Behav*, *31*(2), 143-164. <https://doi.org/10.1177/1090198104263660>

DiClemente, C. C., & Crisafulli, M. A. (2022). Relapse on the Road to Recovery: Learning the Lessons of Failure on the Way to Successful Behavior Change. *J Health Serv Psychol*, *48*(2), 59-68. <https://doi.org/10.1007/s42843-022-00058-5>

Epton, T., Currie, S., & Armitage, C. J. (2017). Unique effects of setting goals on behavior change: Systematic review and meta-analysis. *Journal of consulting and clinical psychology*, *85*(12), 1182-1198. <https://doi.org/10.1037/ccp0000260>

Etkin, J. (2019). Time in relation to goals. *Current Opinion in Psychology*, *26*, 32-36. <https://doi.org/https://doi.org/10.1016/j.copsyc.2018.04.013>

Groshkova, T., Best, D., & White, W. (2013). The Assessment of Recovery Capital: properties and psychometrics of a measure of addiction recovery strengths. *Drug Alcohol Rev*, *32*(2), 187-194. <https://doi.org/10.1111/j.1465-3362.2012.00489.x>

Islam, K. F., Awal, A., Mazumder, H., Munni, U. R., Majumder, K., Afroz, K., Tabassum, M. N., & Hossain, M. M. (2023). Social cognitive theory-based health promotion in primary care practice: A scoping review. *Heliyon*, *9*(4), e14889. <https://doi.org/10.1016/j.heliyon.2023.e14889>

Larimer, M. E., Palmer, R. S., & Marlatt, G. A. (1999). Relapse prevention. An overview of Marlatt's cognitive-behavioral model. *Alcohol Res Health*, *23*(2), 151-160.

Lewis, C. C., Boyd, M., Puspitasari, A., Navarro, E., Howard, J., Kassab, H., Hoffman, M., Scott, K., Lyon, A., Douglas, S., Simon, G., & Kroenke, K. (2019). Implementing Measurement-Based Care in Behavioral Health: A Review. *JAMA Psychiatry*, *76*(3), 324-335. <https://doi.org/10.1001/jamapsychiatry.2018.3329>

Locke, E. A., & Latham, G. P. (2002). Building a practically useful theory of goal setting and task motivation: A 35-year odyssey. *American psychologist*, *57*(9), 705-717. <https://doi.org/10.1037/0003-066X.57.9.705>

Lozano, B. E., & Stephens, R. S. (2010). Comparison of participatively set and assigned goals in the reduction of alcohol use. *Psychology of Addictive Behaviors*, *24*(4), 581-591. <https://doi.org/10.1037/a0021444>

Magill, M., Martino, S., & Wampold, B. E. (2022). Goal setting and monitoring with alcohol and other drug use disorders: Principles and practices. *J Subst Abuse Treat*, *132*, 108650. <https://doi.org/10.1016/j.jsat.2021.108650>

McCurdy, L. Y., Kong, G., Krishnan-Sarin, S., Kiluk, B. D., & Potenza, M. N. (2023). A non-randomized pilot study protocol of a novel social support intervention for individuals in early recovery from hazardous alcohol use. *PLOS ONE*, *18*(10), e0292293. <https://doi.org/10.1371/journal.pone.0292293>

Miller, W. R., & Tonigan, J. S. (1996). Assessing drinkers' motivation for change: The Stages of Change Readiness and Treatment Eagerness Scale (SOCRATES). *Psychology of Addictive Behaviors*, *10*(2), 81-89. <https://doi.org/10.1037/0893-164X.10.2.81>

Ordóñez, L. D., Schweitzer, M. E., Galinsky, A. D., & Bazerman, M. H. (2009). Goals gone wild: The systematic side effects of overprescribing goal setting. *The Academy of Management Perspectives*, *23*(1), 6-16. <https://doi.org/10.5465/AMP.2009.37007999>

Padovano, H. T., Levak, S., Vadhan, N. P., Kuerbis, A., & Morgenstern, J. (2022). The Role of Daily Goal Setting Among Individuals with Alcohol Use Disorder. *Drug Alcohol Depend Rep*, *2*. <https://doi.org/10.1016/j.dadr.2022.100036>

Pearson, E. S. (2012). Goal setting as a health behavior change strategy in overweight and obese adults: a systematic literature review examining intervention components. *Patient Educ Couns*, *87*(1), 32-42. <https://doi.org/10.1016/j.pec.2011.07.018>

Petry, N. M. (2001). Delay discounting of money and alcohol in actively using alcoholics, currently abstinent alcoholics, and controls. *Psychopharmacology*, *154*(3), 243-250.

Schwarzer, R., Jerusalem, M., Weinman, J., Wright, S., & Johnston, M. (1995). Generalized Self-Efficacy Scale. *Measures in Health Psychology: A User's Portfolio. Causal and control beliefs Windsor*.

Skinner, H. A. (1982). The drug abuse screening test. *Addict Behav*, *7*(4), 363-371. <https://doi.org/10.1016/0306-4603(82)90005-3>

Strecher, V. J., Seijts, G. H., Kok, G. J., Latham, G. P., Glasgow, R., DeVellis, B., Meertens, R. M., & Bulger, D. W. (1995). Goal Setting as a Strategy for Health Behavior Change. *Health Education Quarterly*, *22*(2), 190-200. <http://www.jstor.org/stable/45049608>

Substance Abuse and Mental Health Services Administration (2012). SAMHSA’s working definition of recovery: 10 guiding principles of recovery. *https://library.samhsa.gov/product/samhsas-working-definition-recovery/pep12-recdef*

Zimet, G. D., Powell, S. S., Farley, G. K., Werkman, S., & Berkoff, K. A. (1990). Psychometric characteristics of the Multidimensional Scale of Perceived Social Support. *J Pers Assess*, *55*(3-4), 610-617. <https://doi.org/10.1080/00223891.1990.9674095>
